# Effect of Different rhBMP-2 and TG-VEGF Ratios on the Formation of Heterotopic Bone and Neovessels

**DOI:** 10.1155/2014/571510

**Published:** 2014-03-24

**Authors:** Wei Xin Cai, Li Wu Zheng, Chun Lei Li, Li Ma, Martin Ehrbar, Franz E. Weber, Roger A. Zwahlen

**Affiliations:** ^1^Discipline of Oral and Maxillofacial Surgery, Faculty of Dentistry, The University of Hong Kong, Prince Philip Dental Hospital, 34 Hospital Road, Hong Kong; ^2^Discipline of Oral Diagnosis and Polyclinics, Faculty of Dentistry, The University of Hong Kong, Hong Kong; ^3^Discipline of Oral Rehabilitation, Faculty of Dentistry, The University of Hong Kong, Hong Kong; ^4^Department of Obstetrics, University Hospital Zurich, Zurich, Switzerland; ^5^Oral Biotechnology and Bioengineering, Division of Cranio-Maxillo-Facial and Oral Surgery, University Hospital Zurich, Zurich, Switzerland

## Abstract

Bioengineered bone substitutes might represent alternatives to autologous bone grafts in medically compromised patients due to reduced operation time and comorbidity. Due to the lack of an inherent vascular system their dimension is limited to the size of critical bone size defect. To overcome this shortcoming, the experiment tried to create heterotopic bone around vessels. *In vivo*, a two-component fibrin and thrombin gel containing recombinant bone morphogenic protein (rhBMP-2) and transglutamate vascular endothelial growth factor (TG-VEGF) in different ratios, respectively, was injected into a dimensionally stable membrane tube, wrapped around the femoral vessel bundle in twelve New Zealand white rabbits. Sacrifice occurred eight weeks postoperatively. Microcomputed tomography of the specimens showed significantly increased bone volume in the rhBMP-2 to TG-VEGF ratio of 10 to 1 group. Histology showed new bone formation in close proximity to the vessel bundle. Immunohistochemistry detected increased angiogenesis within the newly formed bone in the rhBMP-2 to TG-VEGF ratios of 3 to 1 and 5 to 1. Heterotopic bone was engineered *in vivo* around vessels using different rhBMP-2 and TG-VEGF ratios in a fibrin matrix injected into a dimensionally stable membrane tube which prevented direct contact with skeletal muscles.

## 1. Introduction

Bone substitutes created by tissue engineering provide novel options in the treatment strategy of large bone defects [1]. As engineered bone grafts usually lack inherent vascular systems, promotion of angiogenesis becomes fundamental for their survival once* in situ* [2]. A way to overcome the shortcoming of lacking inherent vascular system in engineered bone could be engineering bone around vessels. Recently,* in vivo* heterotopic bone formation was detected around vessels within a preformed dimensionally stable membrane tube, filled up with a fibrin gel matrix containing bone morphogenetic protein-2 (rhBMP-2) [3].

After heterotopic bone formation around vessels has been detected when administering rhBMP-2 only in different dosages, it became of interest to investigate eventual angiogenesis within this bone, adding vascular endothelial growth factor (VEGF) in different ratios to rhBMP-2. The VEGF family is of utmost importance to induce angiogenesis during bone formation [4]. So far, their interaction with BMP related to bone formation has been widely investigated in animals [5–11]. While endothelial cells modulate osteoprogenitor cell differentiation through a BMP-2 signaling pathway during bone healing [12], endothelial cell proliferation is stimulated by VEGF producing osteoblasts [13]. This interaction, depending on specific BMP to VEGF ratios, was detected in animal models investigating critical bone size defects or ectopic bone formation under direct contact with skeletal muscles; BMP to VEGF ratios in those studies varied widely between 50 : 1 and 1 : 5 [5, 9–11]. The ideal ratio to achieve maximum bone regeneration, however, has not yet been found [5].

TG-VEGF, developed in 2001 [14], was used in this experiment due to its efficient covalent incorporation into fibrin which permits a slow, continuous release and because of its scientifically proven durable promotion in neovessel formation due to its continuous angiogenic signal [15, 16].

The present study investigated heterotopic bone formation with concomitant angiogenesis around vessels promoted by rhBMP-2 to TG-VEGF ratios of 10 : 1, 5 : 1, and 3 : 1, respectively [5, 9–11], in a fibrin matrix, separated from contact with skeletal muscles by a membrane.

## 2. Materials and Methods

### 2.1. Preparation of Tisseel Lyo Containing the Growth Factors

The preparations of the rhBMP-2 and TG-VEGF used in this study have already been described [14, 17, 18]. 125 *μ*g rhBMP-2 (original concentration 1 *μ*g/*μ*L), considered to be the ideal dosage for heterotopic bone formation in a previously performed animal pilot study [3], was diluted in 125 *μ*L Tris-HCl (1 mM) solution. The rhBMP-2 solution was supplemented with 50 *μ*L thrombin (Tisseel Lyo, Baxter, Vienna, Austria) to ultimately obtain 300 *μ*L solution. Three different dosages of TG-VEGF were mixed with fibrinogen, the second component of Tisseel Lyo (Baxter, Vienna, Austria), to create a 75 *μ*L solution. This solution was then diluted in 225 *μ*L Tris-buffered saline (TBS). The fibrinogen and thrombin solutions, each 300 *μ*L, were charged in two different cartridges. These cartridges were mounted into a Duploject syringe (Baxter, Vienna, Austria). On injection, formation of fibrin gel started due to the mixing of both substances within the needle. The process was accomplished* in situ* once injection around the vessels within the Inion guided tissue regeneration (GTR) membrane tube was completed.

### 2.2. Animal Model

The study was approved by the Committee on the Use of Live Animals for Teaching and Research (CULATR no. 1637-08), The University of Hong Kong, Hong Kong.

Twelve New Zealand white rabbits (3.0 to 4.2 kg), 6 to 9 months old, were used in this animal trial. The rabbits were allocated into 4 groups (*n* = 3 per group). The dosage of rhBMP-2 (125 *μ*g) was maintained for all groups. In Group A, only rhBMP-2 (125 *μ*g) was administered. This group served as control group. Groups B, C, and D received different rhBMP-2 to TG-VEGF ratios: 10 to 1 (125 *μ*g to 12.5 *μ*g), 5 to 1 (125 *μ*g to 25 *μ*g), and 3 to 1 (41.7 *μ*g to 12.5 *μ*g), respectively.

All surgical procedures were performed in the Laboratory Animal Unit (LAU) of The University of Hong Kong. Preoperatively 30 mg/kg of long-acting oxytetracycline (Troy Laboratories Pty Limited, Glendenning, Australia) and 30 *μ*g/kg Temgesic (Reckitt Benckiser Health, Slough, UK) were administered. All animals were anesthetized by an experienced veterinarian via injection into the gluteal muscle using a mixture of acepromazine (1 mg/kg; Delvet Pty Ltd., Asquith, Australia), ketamine (45 mg/kg; Alfasan International B.V., Woerden, Holland), and xylazine (5 mg/kg; Alfasan International B.V., Woerden, Holland). The rabbits were put in a supine position with the right knee joint in flexion. A face mask continually delivered 1.5–2% Forane (Halocarbon Laboratories, River Edge, NJ, USA) to maintain adequate anaesthetic depth during the surgery. The animal model in this study corresponds step by step to the one the authors have established and described in detail already elsewhere [3]. In brief, an Inion guided tissue regeneration (GTR) membrane sheet (Inion Oy, Tampere, Finland) was formed into a 1 cm long tube. This tube was wrapped around the femoral vessel bundle and secured in place with two Vicryl 4/0 (Johnson & Johnson, Hong Kong) sutures. After the cylinders were filled up with fibrin gel containing growth factors in various ratios as mentioned above, wound closure was performed in layers with Vicryl 4/0 and Ethilon 5/0 (Johnson & Johnson, Hong Kong) in single suture technique.

Postoperative care was performed in collaboration with veterinarians. Drugs used for postoperative care included long-acting oxytetracycline (30 mg/kg; Troy Laboratories Pty Limited, Glendenning, Australia), buprenorphine (0.05 mg/kg; Reckitt Benckiser Health, Slough, United Kingdom), and meloxicam (0.2 mg/kg; Metacam, Boehringer Ingelheim, Ingelheim, Germany).

### 2.3. Specimen Harvest

Eight weeks postoperatively the animals were sacrificed using pentobarbital sodium (150 mg/kg; Alfasan International B.V., Woerden, Holland). The Inion GTR tubes were dissected together with a surrounding soft tissue pad. Specimens were immersed in 10% neutral formalin solution for histopathological and microcomputed tomography analyses.

### 2.4. Microcomputed Tomography

Each specimen was scanned and reconstructed by a microcomputed tomography machine (SkyScan 1076; Bruker, Kontich, Belgium) with a 0.5 mm aluminum filter, at a voltage of 59 kV, using a current of 149 *μ*A. New bone formation within the Inion GTR cylinders was studied by analyzing serial micro-CT images of the specimen at a 12 *μ*m pixel resolution. The reconstruction data was assessed with CT Analyzer version 1.9 software (SkyScan, Kontich, Belgium). The region of interest (ROI) was specified as the area of bone tissue within the specimen. Bone volume data of the ROI was extracted to perform statistical analysis.

### 2.5. Histologic Examination

The specimens were decalcified in 12.5% ethylenediaminetetraacetic acid (EDTA) for about 2 weeks at a pH 7.2 (Sigma, New York, NY, USA) and room temperature. The solution was changed daily. To avoid any eventual skeletal muscle effect on bone formation at both tube ends, each specimen was cut into three equally sized parts, from which only the middle part was processed for sectioning. Subsequently specimens were dehydrated with graded series of ethanol and embedded in paraffin. With a microtome (Leica RM2155; Leica Biosystems, Nussloch, Germany), five sections, each 6 *μ*m in thickness, were cut in five equally sized pieces of the middle part. All tissue sections were stained with hematoxylin/eosin (H&E) (Sigma, NY, USA; Merck, NJ, USA, resp.) and investigated visually as well as photographed under a light microscope (Leica DMLB), at both ×2 and ×10 magnifications.

### 2.6. Immunohistochemical Staining

Endothelial cell adhesion molecule-1 (CD31) (Abcam, Cambridge, UK), being able to recognize the rabbit-specific signals [19, 20], served as primary antibody to detect microvessels. Tissue sections (6 *μ*m) were deparaffinized in sequential xylene and rehydrated in graded alcohol baths. Sections were rinsed with phosphate buffered saline (PBS, PH 7.4, 0.1 M) (Advanced Technology & Industrial Co., Hong Kong). Antigen retrieval was performed in 0.1% trypsin solution at 37°C for 60 minutes according to manufacturer's instruction. To quench endogenous peroxidase activity, the sections were incubated in freshly prepared 0.3% hydrogen peroxide for 30 minutes. After rinsing in phosphate buffered saline (PBS) for 3 times, the sections were blocked with 1.5% normal goat serum for 60 minutes at 37°C. Thereafter they were rinsed again three times with PBS and incubated overnight in primary mouse antibodies against rabbit CD31 (Abcam, Cambridge, UK) at a dilution of 1 : 20 at 4°C. Subsequently the sections were incubated with anti-mouse immunoglobulin G antibody followed by avidin and biotin reagents for 60 minutes. Diaminobenzidine (DAB, Santa Cruz Biotechnology, Dallas, TX, USA) was used to detect eventual reaction. Finally the sections were then rinsed in deionized water for 5 minutes and counterstained with hematoxylin.

### 2.7. Microvessel Density (MVD) Evaluation

**Figure 1:**
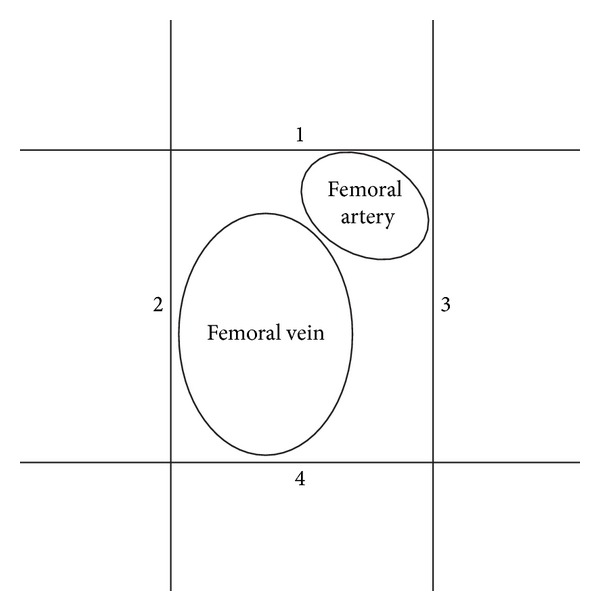
At ×1 magnification, four areas (1–4) were demarcated around the femoral vessel bundle by applying tangents to its borders. Microvessel density in the resulting four areas was counted at ×20 magnification.

After being mounted in a xylene-based medium (VWR International, Radnor, PA, USA), the sections were examined under a light microscope (Leica DMLB; Leica Biosystems, Nussloch, Germany). Microvessel density (MVD) was evaluated according to the guidelines already published elsewhere [21–23]. Positive staining of a single endothelial cell or cluster of endothelial cells was defined as microvessel. Vessels with muscular wall were excluded. The targeted area was defined according to our protocol published previously [20]. Four areas around the femoral vein were determined at ×1 magnification under microscope as indicated in Figure 1. Microvessels in the center of these areas were observed at higher magnification (×20) and counted by a blinded investigator. Mean MVD of these three histological fields was considered as the ultimate evaluation of MVD (microvessel/mm^2^) in one section.

### 2.8. Statistical Analysis

Mean bone volume and MVD among different groups were compared statistically using one-way ANOVA (SPSS version 20, Chicago, IL, USA). The Bonferroni test was used to conduct multiple comparison between each two groups. A *P* ≤ 0.05 was considered to be statistically significant.

## 3. Results 

All animals underwent an uneventful postoperative recovery phase. No adverse events occurred until the time of sacrifice. Two weeks after the operation all animals regained both preoperative nutritional intake and their normal daily activities. Suture removal was performed two weeks postoperatively in all animals, disclosing normal wound healing.

### 3.1. Micro-CT

**Figure 2:**
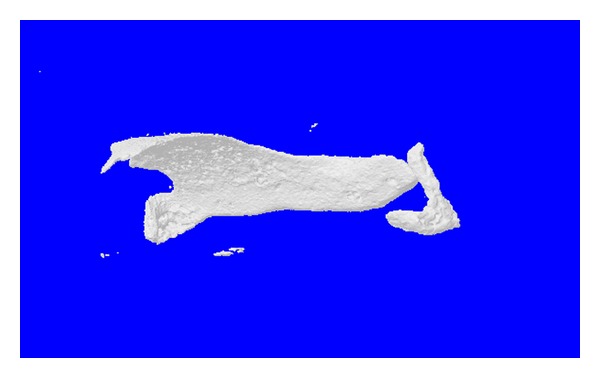
3D micro-CT reconstruction of the newly formed bone within the membrane tube from Group D.

**Figure 3:**
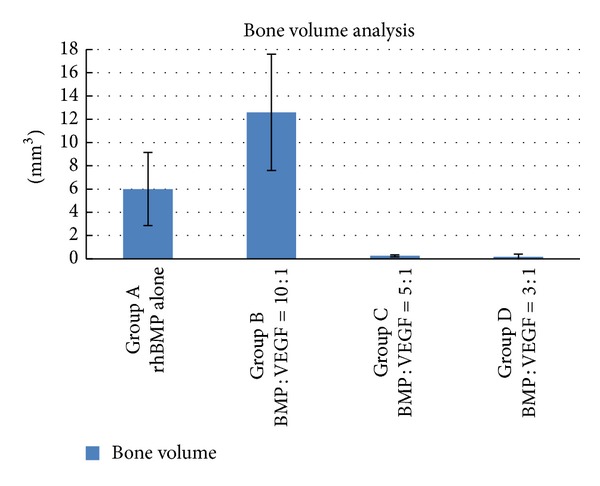
Bone volume amount of the four different groups.

Serial imaging detected newly formed bone tissue in all specimens. 3D reconstruction visualized new bone formation around the vessels (Figure 2). The bone volume in Group B (rhBMP to VEGF is 10 to 1) was significantly increased compared to Groups D (rhBMP to VEGF is 3 to 1) (*P* = 0.005) and C (rhBMP to VEGF is 5 to 1) (*P* = 0.006; Figure 3). Even though quantitatively more bone was found in Group B compared to Group A (rhBMP-2 alone), the difference was not statistically significant (*P* > 0.05; Figure 3).

### 3.2. Histologic Examination

**Figure 4:**
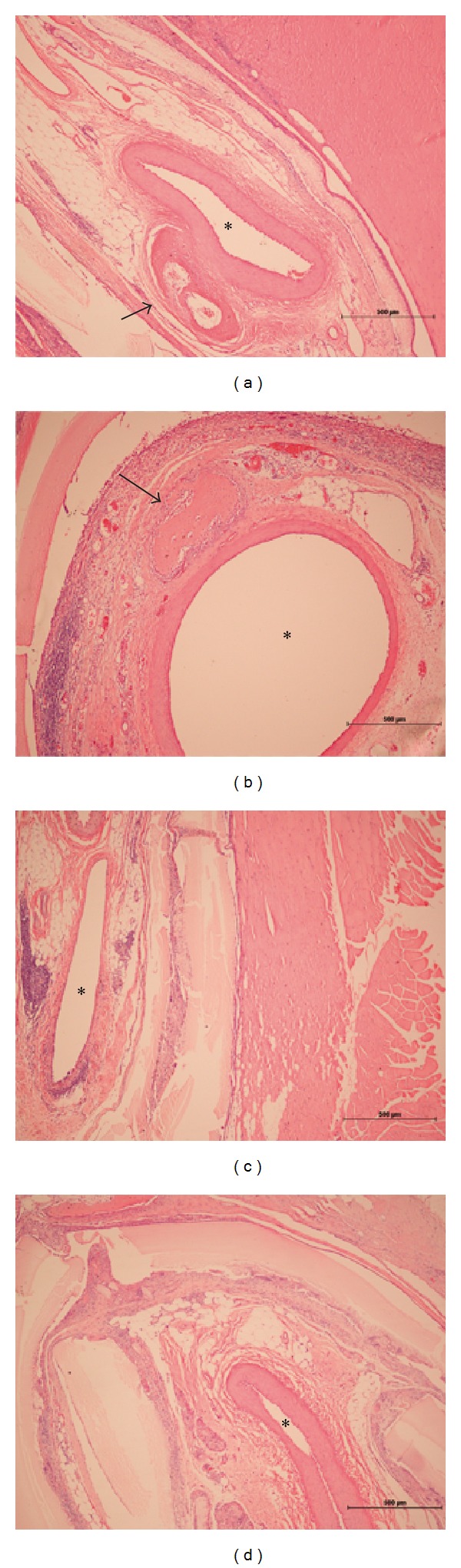
Group A rhBMP-2 alone (a), Group B rhBMP-2 : VEGF = 10 : 1 (b), Group C rhBMP-2 : VEGF = 5 : 1 (c), and Group D rhBMP-2 : VEGF = 3 : 1 (d). Mature bone tissue (arrows) is well visible adjacent to the femoral vessel bundle (∗) in Group A (a) and Group B (b).

Histology reiterated micro-CT findings in terms of newly formed bone tissue in Group D (Figure 4(d)). Most bone tissue was found in proximity to the femoral vessel bundle. Hardly any bone formation was found in Group B and Group C (Figures 4(b) and 4(c)). No bone tissue could be detected near the Inion GTR membrane.

### 3.3. Immunohistochemical Staining

**Figure 5:**
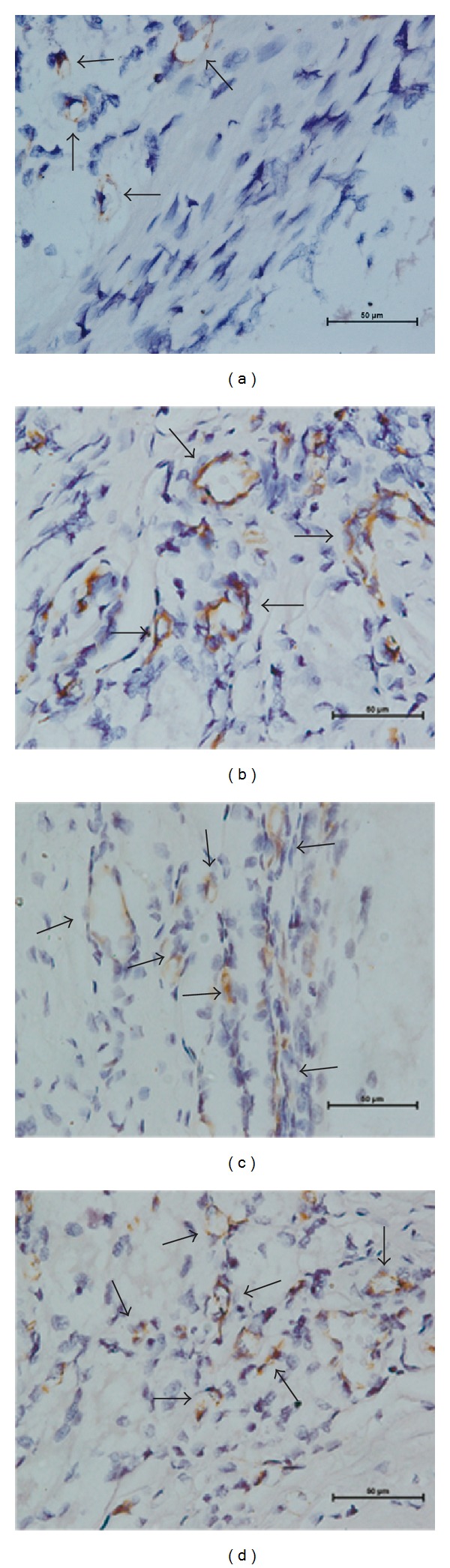
Group A rhBMP-2 alone (a), Group B rhBMP-2 : VEGF = 10 : 1 (b), Group C rhBMP-2 : VEGF = 5 : 1 (c), and Group D rhBMP-2  : VEGF = 3 : 1 (d). Arrows point at microvessels. MVD in groups with both growth factors ((b), (c), and (d)) was increased compared to the group with rhBMP-2 only (a).

Immunohistochemical staining with anti-CD31 antibody visualized clusters of vascular endothelial cells around the femoral vessel bundle (Figures 5(a) to 5(d)). MVD in Group A, Group B, Group C, and Group D was 72.2 ± 3.3, 77.5 ± 8.1, 88.5 ± 5.9, and 85.4 ± 8.5, vessels/mm^2^, respectively. No statistically significant difference among Groups B, C, and D was found regarding MVD.

## 4. Discussion

Deprived of direct skeletal muscle contact, heterotopic bone formation around vessels was detected to be best promoted using the rhBMP-2 to TG-VEGF ratio of 10 to 1. Further on, rhBMP-2 to TG-VEGF ratios of 3 to 1 and 5 to 1, both, increased microvascular density within the newly formed bone.

Interactions of different members of the BMP and VEGF families related to bone development have been investigated in various animal models [5–11, 24]. Many studies [5–10] stated a synergistic or additive effect of both growth factors. However, as mentioned by Young et al., 2009, the ideal ratio of BMP to VEGF depends on particular animal models, study duration, delivery matrixes, and assessment methods [11]. In this study continuous release of growth factors occurred from the fibrin gel being in direct contact to vessels, however, separated from surrounding skeletal muscles by a membrane. Only the rhBMP-2 to TG-VEGF ratio of 10 to 1 seemed to promote heterotopic bone formation. Further on, the bone volume at low rhBMP-2 to TG-VEGF ratios was less than in the group with rhBMP-2 alone, a finding which is in line with the results of Peng et al. in 2005 and 2002 [9, 10]. They found impaired bone formation in decreased BMP to VEGF ratios and concluded that increased proportions of VEGF probably led mesenchymal stem cells to differentiate into endothelial rather than osteogenic cells. A similar finding was revealed in this study, where increased MVD was detected in groups with decreased rhBMP-2 to TG-VEGF ratios.

TG-VEGF contains an additional factor XIIIa substrate sequence. It was developed in 2001 by Zisch et al. [14]. Its highly efficient covalent incorporation into fibrin, preventing its rapid clearance and allowing an increased vessel induction effect compared to the wild type VEGF, was confirmed* in vitro* [15]. Since 2008, its durable promotion in neovessel formation due to the release of a continuous angiogenic signal has been approved scientifically [16].

The Inion GTR membrane tube provided two distinct functions: (1) it prevented direct contact between the fibrin matrix loaded with the growth factors and the surrounding skeletal muscles, and (2) it served as a scaffold for the fibrin gel, as it became dimensionally stable when hardened. This dimensional stability of the tube provided the basis for the steady release of rhBMP-2 and TG-VEGF from the fibrin gel, probably resulting in the formation of bone and microvessels.

Today's CAD/CAM technique allows for the generation of custom made 3D anatomically identical bone pieces out of different materials for immediate bone substitution. Load bearing biodegradable material might become a real alternative in bone reconstructive procedures. They could be of help to reduce operation time and therefore the comorbidity due to autologous microvascular composite grafts, especially in medically compromised patients. Ideally biodegradable material should be substituted by in-growing bone from the contact zones between material and local bone. In larger than critical bone size defects, however, it is still debated whether this process occurs late or not at all in the center of bone grafts. The here presented heterotopic bone formation around vessels possibly could represent a method to overcome this problem. What if a biodegradable, load bearing material could be used as a gouged scaffold where a close-by dissected or mobilized vessel bundle could be laid inside and covered with fibrin gel containing the above-mentioned growth factors? Heterotopic bone engineering around vessels is an interesting concept to further investigate.

The following critical shortcomings need to be pointed out. First, MVD among the groups with various rhBMP-2 and TG-VEGF ratios turned out not to be of statistical significance, similar as the amount of bone generation between Groups A and D. This lack of significance originates most probably from the small animal number. Second, to exclude preexistent vessels in the specimen, vessels with a muscle layer were not counted when assessing the MVD. As sacrifice occurred in the postoperative week eight, some TG-VEGF elicited, but already more mature vessels therefore might have been left out of being counted, biasing the end result.

Third, to fill the Inion GTR membrane tube with the fibrin gel emerged as a technical shortcoming. A temporary hand glove drain had to be used to close one end of the tube during the filling procedure, a somewhat difficult and imprecise task.

Future research with this animal model and larger sample sizes should focus on the investigation of biodegradable and load bearing scaffolds, such as boron hardened bioglass. With this material sacrifice might be performed at 12 weeks or later, a time span not feasible for the here used membrane with its degradation time of 8 weeks. Further on, investigations related to bone formation with angiogenesis should target rhBMP-2 to TG-VEGF ratios between 5 to 1 and 10 to 1.

## 5. Conclusions

Heterotopic bone was created around vessels without direct contact to skeletal muscle. Synergistic effect of rhBMP-2 and TG-VEGF related to bone formation depended on their particular ratios. Whereas a ratio of 10 to 1 turned out to be in favor for bone formation, angiogenesis was promoted using ratios of 3 to 1 and 5 to 1, respectively.
